# Editorial: Advances in Pollen Research: Biology, Biotechnology, and Plant Breeding Applications

**DOI:** 10.3389/fpls.2022.876502

**Published:** 2022-03-29

**Authors:** Concepción Gómez-Mena, David Honys, Raju Datla, Pilar S. Testillano

**Affiliations:** ^1^Department of Plant Development and Hormone Action, Instituto de Biología Molecular y Celular de Plantas (CSIC-Universitat Politècnica de València), Ciudad Politécnica de la Innovación, Valencia, Spain; ^2^Laboratory of Pollen Biology, Institute of Experimental Botany of the Czech Academy of Sciences, Prague, Czechia; ^3^Global Institute for Food Security, University of Saskatchewan, Saskatoon, SK, Canada; ^4^Pollen Biotechnology of Crop Plants Group, Margarita Salas Center of Biological Research, CIB -CSIC, Madrid, Spain

**Keywords:** pollen, anther, biotechnology, breeding, crops

In flowering plants, mature pollen grains are produced within the developing anthers of the flowers in two successive phases, microsporogeneseis and microgametogenesesis. Anther and pollen development involves the coordinated growth and differentiation of tissues and cell types required for the formation of viable male gametes, but also, to permit pollen release and ensure successful fertilization. The identification of the genetic networks and regulatory molecules involved in the formation of the anther remains a highly unexplored topic in many crops. Evolutionary and functional studies in crops are helping to unravel new functions for genes originally identified in model plants (Rojas-Gracia et al., 2017) and the changes in transcriptional profiles associated with plant domestication (Xiang et al., 2019). Under the global changes in environmental conditions, pollen development is probably one of the most vulnerable and challenged stages of plant reproduction (Chaturvedi et al., 2021). In this context, increasing basic research will provide valuable information to assist the design of biotechnological tools to mitigate this effect especially in crop systems in the future.

New advances in the functional characterization of genes involved in different aspects of pollen development and function are described in the following articles of this Research Topic. In the original research article by Hamza et al., the authors functionally characterized *Pisum sativum ENDOTHECIUM* 1 (*PsEND1*), a pea anther-specific gene that encodes a protein containing four hemopexin domains. Gain and loss-of-function experiments showed that PsEND1 is a central player in the maintenance of balanced redox levels during pollen and anther development. Zhou L. et al. demonstrated pollen-specific regulatory function of ZmLarp6c1, maize ortholog of Arabidopsis AtLARP6C, particularly during the progamic phase. The respective Ds-GFP transposable element insertion line showed reduced transmission through the male wich was associated with altered germination dynamics and slower growth of mutant pollen tubes. Considering that LARP6C also orchestrates posttranscriptional reprogramming of gene expression during hydration in Arabidopsis (Billey et al., 2021), these findings highlight the general regulatory role of LARP6C in angiosperms. The original research article by Mazuecos-Aguilera et al. describes the functional study of the *INAPERTURATE POLLEN1* (*INP1*) ortholog from the basal eudicot *Eschscholzia californica* (California poppy), a gene involved in pollen aperture formation. This study substantiates the importance of *INP1* homologs for aperture formation across angiosperms and opens up new avenues for functional studies of other aperture candidate genes. The role of the Arabidopsis Ankyrin-repeat protein (AT5G66055, AKRP), during male and female gametogenesis is analyzed by Kulichová et al. using the new mutant allele *akrp-3*. AKRP is a plastid-localized protein with a putative function in plastid differentiation and morphogenesis. The findings provide insight into the role of this protein in both the differentiation of gametophytes and the coupling of embryo development with chlorophyll synthesis. In the research article by Dong et al., the authors reported a new major gene *Cla006625* controlling “Genic Male Sterility” (GMS) in watermelon. Molecular and genomics studies revealed that this gene renamed as *ClaPEX1* encodes a leucine-rich repeat protein, and the recessive mutant of this locus causes pollen abortion conferring GMS. The targeted RNAi based evidence further confirmed the functionality for this gene in GMS, and the authors propose potential applications in hybrid seed production technology to capture heterosis in watermelon. Kakui et al. studied pollen number variation in Arabidopsis and discovered the first gene responsible for pollen number, *REDUCED POLLEN NUMBER1* (*RDP1*), encoding the large ribosomal subunit assembly factor. CRISPR/Cas9-generated *rdp1-3* mutants revealed the pleiotropic effect of RDP1 in flowering and pollen development. Subsequent transcriptome analysis supported the hypothesis that ribosome biogenesis, critical for pollen development, is disturbed in the *rdp1-3* mutant pollen and highlighted three key bHLH transcription factors (ABORTED MICROSPORES, bHLH010, and bHLH089).

The identification and characterization of regulatory molecules during anther or pollen development is the subject of the remaining articles in this Research Topic. The role of hormone dynamics during microgametogenesis has been explored by Záveská Drábková et al. in several *Nicotiana* species. The article describes the dynamic changes in endogenous phytohormones during pollen ontogeny, highlighting that unequal levels of endogenous hormones and the presence of specific derivatives which may be characteristic for pollen development in different phylogenetic plant groups. In the original research article by Zhou D. et al., the author used male-sterile systems of *Brassica campestris* (Chinese cabbage) to study anther and pollen development in this species. Differentially expressed lncRNAs (DELs), miRNAs (DEMs), and genes (DEGs) were identified providing new insights into molecular regulation especially the ncRNA interaction during pollen development in Brassica crops.

Several of the articles submitted to this Research Topic investigated global developmental aspects of pollen development and its impact on plant performance. Xue et al., developed several live imaging methods for the study of anther development. They created the marker line *ProUBQ10:H2B:VENUS* and used it to study the development of the middle layer in the anther of *Arabidopsis thaliana*. The results showed that the middle layer was derived from both inner and outer secondary parietal cells, indicating that the cell fate determination of the middle layer was non-cell-autonomous in Arabidopsis. In the new research report by Xiao et al., the authors investigated the adaptive and evolutionary features of “Delayed Autonomous Selfing” (DAS) in *Salvia umbractica*. The observations and findings from the field and controlled experiments showed outcrossing using insect pollinators first, wich failed to fertilize, lead to execution of DAS to ensure fertilization for successful fruit and seed production. The authors presented strong supporting evidence by detailed documentation of changes in the reproductive organs' specific behaviors linked with morphological and developmental activities of these two built-in alternative pollination options in *Salvia* species. In the research article by Calić et al., the authors investigated the influence of long-term storage temperature on pollen viability of four Serbian autochthon apple cultivars. Interestingly, the pollen could be efficiently maintained at −20°C and later used for breeding purposes. The results will surely contribute to the preservation of these old autochthon cultivars as unique genetic resources with important ecological and economic value. Jaffri and MacAlister utilized histology and immunostaining to show the structure of the tomato pollen wall, characterized dynamic changes in pectin composition, and established a developmental timeline of its formation. Following meiosis, the microspores losing their cellulose primary wall remain connected by a temporary callose wall in tetrads. Release of early microspores initiates sporopollenin secretion to form exine, wich is completed in late microspores. The tomato pollen wall formation is finished by the formation of intine from pollen mitosis I to pollen maturation. Grienenberger and Quilichini highlighted significant progress in the field of sporopollenin research; they examined the cross-disciplinary efforts to solve the sporopollenin composition puzzle and presented a working model of sporopollenin's molecular structure and biosynthesis. They further discussed the controversies and remaining knowledge gaps, including the degree of aromaticity, cross-linkage profiles, and extent of chemical conservation of sporopollenin among land plants. Finally, the authors highlighted opportunities for practical utilization of this extraordinary biomaterial.

Pollen biotechnology offers a wide range of possibilities for plant breeding. Doubled-haploid technology, based on the reprogramming of immature pollen grains or microspores toward embryogenesis, promises to accelerate crop breeding programs and shorten the time to obtain new varieties. Investigations in recent years have shown the complex regulatory mechanisms underlying microspore embryogenesis (Testillano, 2019), opening promising avenues for improving its efficiency in crop species of economic and environmental interest. In addition, genetically engineered male-sterile plants offer a valuable trait for plant breeding programs for many crops. Recently, CRISPR/Cas9 editing technology has become an efficient and versatile option to obtain new plant varieties and accelerate breeding practices. In the review by  et al., the authors recapitulated past and present research on obtaining male-derived haploid progeny by microspore embryogenesis. The authors evaluated basic breeding applications of this process, explored the utility of genomics and gene editing technologies for protocol development, and provided considerations to overcome genotype specificity and morphogenic recalcitrance in non-model plant systems. In the article by Pandey et al., the authors reviewed the molecular mechanisms controlling the alternation of generations between the sporophytic and gametophytic stages from an evolutionary perspective. The article compares the genetic factors and mechanisms regulating the separation of the two developmental programs and discusses its biotechnological applications for accelerating the breeding of crop plants.

The goal of this Research Topic was to highlight the latest advances in pollen research and the potential of pollen in the development of biotechnological applications for plant breeding. Fifteen articles have been published on this Research Topic including two reviews, one mini-review, and 12 original research articles covering different aspects of pollen biology, biotechnology, and breeding applications. Remarkably, many of the research articles were carried out on important crops including studies in apple, pea, maize, watermelon, or tomato. Globally this Research Topic of articles successfully represents some major advances in pollen research across different plant species contributing to increasing knowledge in the field and to the generation of new opportunities to implement crop improvement programs in the coming years.

## Author Contributions

All authors listed have made a substantial, direct, and intellectual contribution to the work and approved it for publication.

## Conflict of Interest

The authors declare that the research was conducted in the absence of any commercial or financial relationships that could be construed as a potential conflict of interest.

## Publisher's Note

All claims expressed in this article are solely those of the authors and do not necessarily represent those of their affiliated organizations, or those of the publisher, the editors and the reviewers. Any product that may be evaluated in this article, or claim that may be made by its manufacturer, is not guaranteed or endorsed by the publisher.
